# Genome-wide identification and functional analyses of calmodulin genes in *Solanaceous* species

**DOI:** 10.1186/1471-2229-13-70

**Published:** 2013-04-27

**Authors:** Yuan Zhao, Wei Liu, You-Ping Xu, Jia-Yi Cao, Janet Braam, Xin-Zhong Cai

**Affiliations:** 1 Institute of Biotechnology, College of Agriculture and Biotechnology, Zhejiang University, 866 Yu Hang Tang Road, Hangzhou 310058, China; 2 Center of Analysis and Measurement, Zhejiang University, 866 Yu Hang Tang Road, Hangzhou 310058, China; 3 Department of Biochemistry and Cell Biology, Rice University, Houston, TX 77005-1892, USA

**Keywords:** Calcium, Calmodulin, Gene Structure, Phylogenetic Analysis, Defense, Resistance, Tomato, *Nicotiana Benthamiana*, Potato

## Abstract

**Background:**

Calmodulin (CaM) is a major calcium sensor in all eukaryotes. It binds calcium and modulates the activity of a wide range of downstream proteins in response to calcium signals. However, little is known about the *CaM* gene family in *Solanaceous* species, including the economically important species, tomato (*Solanum lycopersicum*), and the gene silencing model plant, *Nicotiana benthamiana*. Moreover, the potential function of CaM in plant disease resistance remains largely unclear.

**Results:**

We performed genome-wide identification of *CaM* gene families in *Solanaceous* species. Employing bioinformatics approaches, multiple full-length *CaM* genes were identified from tomato, *N. benthamiana* and potato (*S. tuberosum*) genomes, with tomato having 6 *CaM* genes, *N. benthamiana* having 7 *CaM* genes, and potato having 4 *CaM* genes. Sequence comparison analyses showed that three tomato genes, *SlCaM3/4/5*, two potato genes *StCaM2/3*, and two sets of *N. benthamiana* genes, *NbCaM1/2/3/4* and *NbCaM5/6,* encode identical CaM proteins, yet the genes contain different intron/exon organization and are located on different chromosomes. Further sequence comparisons and gene structural and phylogenetic analyses reveal that *Solanaceous* species gained a new group of *CaM* genes during evolution. These new *CaM* genes are unusual in that they contain three introns in contrast to only a single intron typical of known *CaM* genes in plants. The tomato *CaM* (*SlCaM*) genes were found to be expressed in all organs. Prediction of cis-acting elements in 5' upstream sequences and expression analyses demonstrated that *SlCaM* genes have potential to be highly responsive to a variety of biotic and abiotic stimuli. Additionally, silencing of *SlCaM2* and *SlCaM6* altered expression of a set of signaling and defense-related genes and resulted in significantly lower resistance to *Tobacco rattle virus* and the oomycete pathogen, *Pythium aphanidermatum.*

**Conclusions:**

The *CaM* gene families in the *Solanaceous* species tomato, *N. benthamiana* and potato were identified through a genome-wide analysis. All three plant species harbor a small set of genes that encode identical CaM proteins, which may manifest a strategy of plants to retain redundancy or enhanced quantitative gene function. In addition, *Solanaceous* species have evolved one new group of *CaM* genes during evolution. *CaM* genes play important roles in plant disease resistance to a variety of pathogens.

## Background

Calcium (Ca^2+^) is an essential element in plant cell wall and an important nutrient for plant growth. In addition, Ca^2+^ acts as a second messenger to regulate a variety of biological processes in response to various biotic and abiotic stimuli in eukaryotic organisms [1-4].

Calmodulin (CaM) is a major Ca^2+^ sensor thought to interpret Ca^2+^ signatures in plants. It is a small protein, typically comprising only about 149 amino acids. It bears four helix-loop-helix motifs called EF hands, each with the ability to bind Ca^2+^[5].

*CaM* genes have been identified in several plant species. Genome-wide identification of *CaM* genes in model plant species, such as *Arabidopsis* and rice [5-7], has revealed that CaM proteins are typically encoded by gene families. In addition, plants may contain several CaM isoforms that differ in only a few amino acids, with one of the isoforms being encoded by several genes located on different chromosomes of the genome. For example, in *Arabidopsis*, seven genes encode for four CaM isoforms, among which *CaM1* and *CaM4* encode an identical protein sequence and *CaM2*, *CaM3* and *CaM5* also encode an identical protein sequence, differing in only a few amino acids from the CaM1/CaM4 isoform. The four *Arabidopsis* CaM isoforms differ from each other in only one to five amino acids [5]. In rice, five genes encode three CaM isoforms. *OsCaM1-1*, *OsCaM1-2* and *OsCaM1-3* encode an identical protein sequence. OsCaM2 and OsCaM3 proteins have only two amino acid differences. In addition, *CaM* genes have been identified in other plant species, such as tobacco [8], potato [9] and soybean [10]. Nevertheless, CaMs in many *Solanaceous* species, including the economically important species tomato and the model species for gene silencing studies, *Nicotiana benthamiana*, have not yet been identified or characterized. Genome-wide analysis of *CaM* gene families in other *Solanaceous* species has also not yet been conducted.

As major Ca^2+^ sensors, CaMs are multifunctional in plants. CaMs play important roles in regulation of growth, development and abiotic stress resistance in plants [11,12]. For example, over-expression of *AtCaM7* in *Arabidopsis* promotes photomorphogenetic growth [13]. A loss-of-function mutation in *AtCaM2* causes a significant reduction in pollen germination [14]. In marigold, CaM regulates adventitious root development [15]. Over-expression of *OsCaM1*, *MCaM3* and *GmCaM4* in rice, mulberry and soybean, respectively, enhances resistance to drought and/or salt [16-19].

Limited evidence has been reported for a role of CaM in plant disease resistance. Ectopic over-expression of the soybean *CaM* genes, *SCaM4* and *SCaM5*, enhances resistance to *Phytophthora parasitica* var. *nicotianae*, *Pseudomonas syringae* pv. *tabaci* and TMV in transgenic tobacco [20] and to *P. syringae* pv. *tomato* DC3000 in transgenic *Arabidopsis*[21]. Over-expression of *CaCaM1* promotes reactive oxygen species (ROS) and nitric oxide (NO) generation, and increases resistance to *Xanthomonas campestris* pv. *vesicatoria* in pepper [22]. Knockdown of *NtCaM13* expression enhances susceptibility to *Ralstonia solanacearum* and *Rhizoctonia solani* in tobacco [23]. CaM may directly bind with CAMTA3/SR1, which binds to and negatively regulates *EDS1*, and thus down-regulate salicylic acid dependent defense and resistance [24]. Different isoforms of a CaM family may play various roles in the regulation of plant defense [25].

Taking advantage of the recent release of complete genomes of a number of *Solanaceous* species, we performed a genome-wide identification of *CaM* gene families in tomato, *N. benthamiana* and potato. Through systemic phylogenetic, gene structure and expression analyses, we discovered one novel group of *CaM* genes in *Solanaceous* species, and demonstrated that a small set of genes encode an identical CaM protein sequence, as may be typical of plants [5-7]. Multiple genes encoding identical proteins may ensure redundancy for a critical life function or may be needed to produce sufficient protein product. Alternatively, multiple *CaM* genes may be evidence of a strategy of plants to efficiently evolve functional gene paralogs. Finally, we provide evidence that reveals function for SlCaMs in resistance to viral and oomycete pathogens.

## Results

### Identification of *CaM* genes in *Solanaceous* genomes

**Figure 1:**
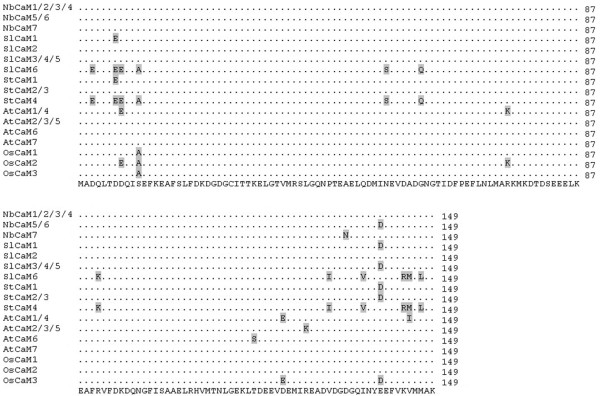
**Alignment profile of CaM proteins of *Solanaceous *species, *Arabidopsis *and rice.** The accession numbers for CaM proteins of tomato, potato and *Nicotiana benthamiana* were listed at Table 1 while those for *Arabidopsis* and rice CaM proteins were as follows: AtCaM1 (AT5G37780.1), AtCaM2 (AT2G41110.1), AtCaM3 (AT3G56800.1), AtCaM4 (AT1G66410.1), AtCaM5 (AT2G27030.1), AtCaM6 (AT5G21274.1), AtCaM7(AT3G43810.1), OsCaM1 (LOC_Os03g20370.1, LOC_Os07g48780.1, LOC_Os01g16240.1), OsCaM2(LOC_Os05g41210.1), OsCaM3(LOC_Os01g17190.1).

**Figure 2:**
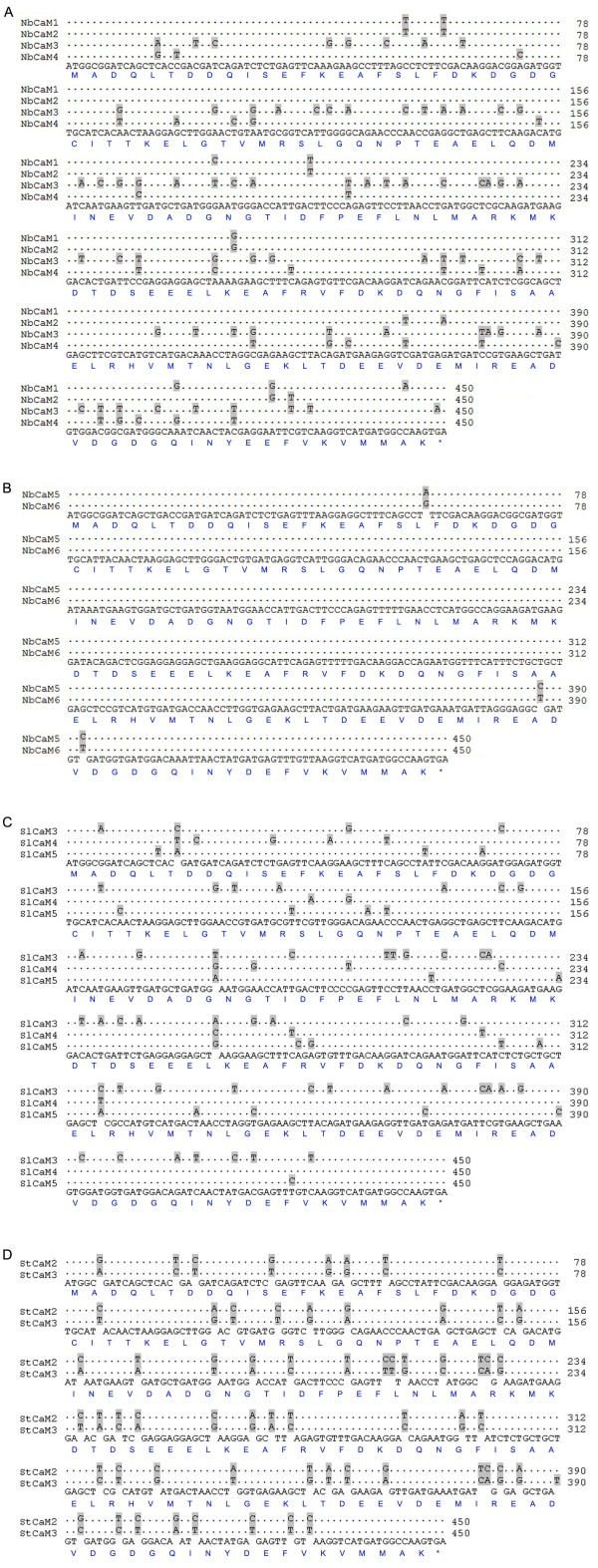
**Alignment profile of the *Solanaceous CaM *genes that encode an identical protein isoform.** Coding sequences of *NbCaM1/2/3/4* **(A)**, *NbCaM5/6* **(B)**, *SlCaM3/4/5* **(C)** and *StCaM2/3* **(D)** were aligned. The corresponding amino acid sequences are also shown.

To identify *CaM* genes in genomes of *Solanaceous* species, all four *Arabidopsis* and three rice CaM protein sequences were collected and used for TBLASTN search against the databases from SGN (http://solgenomics.net/). Seventy three, 96 and 81 nonreduntant sequences were retrieved in tomato, potato (*S. tuberosum*) and *Nicotiana benthamiana* genomes, respectively. These sequences were aligned with the canonical *Arabidopsis* CaM (AtCaM2) with CLUSTALX program and viewed by GeneDoc for the sequence identity to AtCaM2. Sequences were further analyzed by Pfam (http://pfam.sanger.ac.uk/) and CDD (http://www.ncbi.nlm.nih.gov/cdd) programs to confirm presence of the EF-hand domains, a characteristic domain for Ca^2+^ binding. Genes with sequence identity of over 90% to AtCaM2 and that harbored four EF-hands were defined as CaMs, following precedent set previously [5]. The genomic, cDNA and protein sequences were comparatively analyzed for all these CaM candidates. We found that the cDNA and protein sequences of one of the *N. benthamiana* CaMs (NbS00037851g0005.1) previously deposited in the database were incorrect due to an error in identification of the second intron. The sequences have been corrected (Table 1, Figures 1 and 2). Finally, six, four and seven full-length *CaM* genes were indentified in tomato, potato and *N. benthamiana* genomes. To better reflect the orthologous relationship between the *Solanaceous* and *Arabidopsis CaM* genes, we named the *Solanaceous CaM* members in accordance with their phylogenies and sequence similarity to individual *AtCaMs* (Table 1).

**Table 1:** **The *CaM *gene families in *Solanaceous *species**

**CaM Gene**	**Locus number**^**1**^	**Chromosome/location**	**EF hands**	**Protein size (aa)**	**Mol Wt (kDa)**	**pI**	**Intron**	**% of Met**	**Cys27**^**2**^	**K116**^**3**^	**% amino acid identity to canonical CaM**
SlCaM1	Solyc01g008950.2.1	ch1/2963201-2960056	4	149	16.85	3.95	1	6.0	+	+	98%
SlCaM2	Solyc10g081170.1.1	ch10/61628347-61626964	4	149	16.85	3.95	1	6.0	+	+	99%
SlCaM3	Solyc10g077010.1.1	ch10/59260504-59262472	4	149	16.83	3.93	1	6.0	+	+	Cannonical SlCaM
SlCaM4	Solyc11g072240.1.1	ch11/52540732-52538666	4	149	16.83	3.93	1	6.0	+	+	Cannonical SlCaM
SlCaM5	Solyc12g099990.1.1	ch12/65252546-65249443	4	149	16.83	3.93	1	6.0	+	+	Cannonical SlCaM
SlCaM6	Solyc03g098050.2.1	ch3/53844509-53847335	4	149	16.93	4.00	3	6.0	+	+	91%
NbCaM1	NbS00008025g0011.1	-	4	149	16.85	3.95	1	6.0	+	+	Cannonical NbCaM
NbCaM2	NbS00010343g0006.1	-	4	149	16.85	3.95	1	6.0	+	+	Cannonical NbCaM
NbCaM3	NbS00055418g0001.1	-	4	149	16.85	3.95	1	6.0	+	+	Cannonical NbCaM
NbCaM4	Corrected form of NbS00037851g0005.1	-	4	149	16.85	3.95	1	6.0	+	+	Cannonical NbCaM
NbCaM5	NbS00051963g0003.1	-	4	149	16.83	3.93	1	6.0	+	+	99%
NbCaM6	NbS00061039g0001.1	-	4	149	16.83	3.93	1	6.0	+	+	99%
NbCaM7	NbS00041363g0012.1	-	4	149	16.85	3.98	1	6.0	+	+	99%
StCaM1	PGSC0003DMC400039343	-	4	149	16.85	3.95	1	6.0	+	+	99%
StCaM2	PGSC0003DMC400047620	-	4	149	16.83	3.93	1	6.0	+	+	Cannonical StCaM
StCaM3	PGSC0003DMP400012777	-	4	149	1.683	3.93	1	6.0	+	+	Cannonical StCaM
StCaM4	PGSC0003DMP400056198	-	4	149	1.693	4.00	3	6.0	+	+	91%

^1^ NbCaM4 was deduced from the genomic sequence Niben044Scf00037851 and was the corrected form of NbS00037851g0005.1.

^2^ Presence of a cysteine at the position of 27 in a CaM protein was indicated as a “+”.

^3^ Presence of a lysine at the position of 116 in a CaM protein was indicated as a “+”.

Sequence analyses showed that all *Solanaceous* CaM proteins are comprised of 149 amino acids (Table 1). Motif analysis using Pfam and CDD revealed that all of the *Solanaceous* CaMs carry two pairs of EF-hand domain, corresponding to two EF-hand 7 motifs (PF13499). Sequence alignment using ClustalX demonstrated that members of the *Solanaceous* CaM families are highly conserved in amino acid sequence, with over 90% sequence identity (Figure 1). The tomato CaMs encoded by *SlCaM3*, *SlCaM4* and *SlCaM5* are identical in amino acid sequence while the other three SlCaM proteins share 91%-99% amino acid sequence identity with SlCaM3/4/5. The potato StCaM2 and StCaM3 protein sequences are identical while StCaM1 and StCaM4 share 99% and 91% amino acid identity with StCaM2/3, respectively. In the case of *N. benthamiana* CaMs, *NbCaM1*, *NbCaM2*, *NbCaM3*, and *NbCaM4* encode an identical protein sequence, and *NbCaM5* and *NbCaM6* encode an identical protein also. The other two NbCaMs exhibit 99% sequence identity to NbCaM1/2/3/4 (Table 1, Figure 1). However, nucleotide sequences of the *CaM* gene families are much more diverse in comparison with protein sequences. The identity percentage of nucleotide sequences within a *CaM* gene family is 79%-92% for *SlCaMs*, 79%-91% for *StCaMs* and 83%-98% for *NbCaMs*, respectively (Additional file 1). Notably, even the genes that encode identical CaM protein sequences are not identical. For example, the coding sequences of *SlCaM3/4/5*, *StCaM2/3*, *NbCaM1/2/3/4* and *NbCaM5/6* share only 88%-92%, 86%, 83%- 96% and 99%, respectively (Figure 2). These genes differ primarily in the third nucleotide of the amino acid coding triplets (Figure 2).

In addition to the *CaM* genes described above, 67, 77 and 89 other sequences of tomato, potato and *N. benthamiana* respectively, were retrieved from BLAST searches using *Arabidopsis* and rice CaMs (Additional file 2). Of these sequences, 33, 46 and 55 sequences of tomato, potato and *N. benthamiana* respectively, are shorter than 200 amino acids long and contain EF-hands but no any other known functional domain. These protein sequences were thus designated as *Solanaceous* calmodulin-like (CML) sequences, following the criteria used previously [4]. Of the remaining sequences, 29 tomato, 30 potato and 34 *N. benthamiana* sequences were longer than canonical CaMs, having more than 500 amino acids, and harbor not only EF-hand domains but also protein kinase domain(s); these may be calcium-dependent protein kinase candidates.

### Gene structure and chromosome location of *Solanaceous CaM* genes

**Figure 3:**
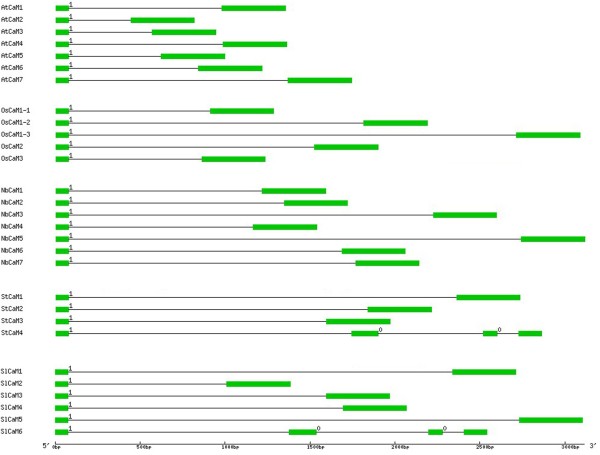
**Schematic diagram representing structures of *CaM *genes of *Solanaceous *species, *Arabidopsis *and rice.** The accession numbers for *CaM* genes are listed in Figure 1 legend. Exons and introns are indicated as green boxes and black lines, respectively. Intron phase numbers 0 and 1 are also shown at the beginning of the introns. The diagram is drawn to scale.

The finding that several genes encode identical CaM proteins in a variety of *Solanaceous* species prompted us to investigate gene structure and chromosome location of these *CaM* genes in the different species. Results of comparisons of genomic DNA sequences with corresponding cDNA sequences showed that coding sequences of *CaM* genes are interrupted by introns. It is noteworthy that unlike *Arabidopsis CaM* genes, which carry a single intron, the number of introns varied in *Solanacous CaM* genes, with one in *SlCaM1* to *SlCaM5*, *StCaM1* to *StCaM3* and all seven *NbCaMs*, and three introns in *SlCaM6* and *StCaM4* (Table 1 and Figure 3). Interestingly, all *Solanaceous CaM* genes were disrupted by the first intron in the Gly26 codon, similar to that previously reported for *Arabidopsis CaMs*[5]. For those genes that contain 3 introns, the interruption sites by the 2nd and 3rd introns were Asp70 and Leu106 codons, respectively. The intron that interrupted Gly26 codon was phase 1 type, while the other two belonged to phase 0 type (Figure 3). In addition, generally, the sizes of the introns are significantly longer in *Solanaceous CaMs* compared with *Arabidopsis CaMs*, but are similar in length to rice *CaMs* (Figure 3).

Chromosome localization analysis demonstrated that the *SlCaM* genes are located on different chromosomes of the tomato genome. Both *SlCaM2* and *SlCaM3* are located on chromosome 10, while the other four *SlCaM* genes are on four different chromosomes (1, 3, 11 and 12), respectively (Table 1). Information about the chromosome location of *CaMs* in other *Solanaceous* species is not yet available.

### Phylogenetic relationship among *Solanaceous* and *Arabidopsis* and rice CaM proteins

**Figure 4:**
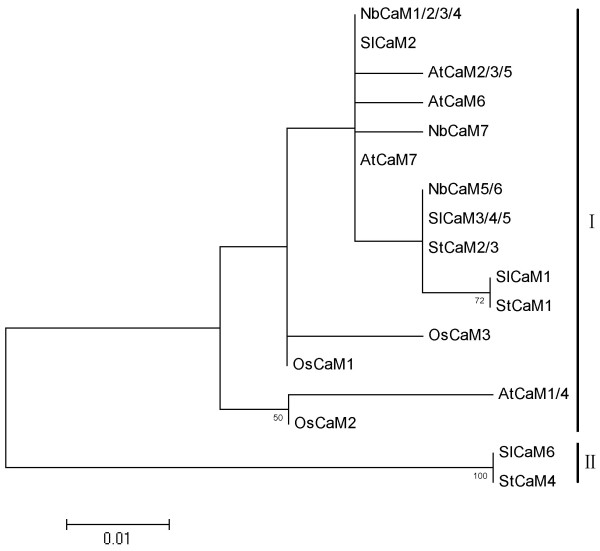
**Phylogenetic tree for CaM proteins of *Solanaceous *species, *Arabidopsis *and rice.** A CaM protein that is encoded by more than one gene is named as a combination of all the gene numbers. The accession numbers for the CaMs are listed in  Figure 1 legend.

The full-length amino acid sequences of *Solanaceous* and *Arabidopsis* and rice CaMs were subjected to phylogenetic analysis. A maximum likelihood (ML) phylogenetic tree was constructed. The CaM proteins clustered into two major groups (Figure 4). Most *Solanaceous* CaMs belonged to the same group, named Group I here. These included 5 out of 6 SlCaMs; 3 out of 4 StCaMs and all 7 NbCaMs. All five *Arabidopsis* and three rice CaMs also belonged to Group I. However, two *Solanaceous* CaMs, SlCaM6 and StCaM4 segregated into another group (Group II) (Figure 4). It is intriguing that the *Solanaceous*, *Arabidopsis* and rice CaMs formed different groups. All *Arabidopsis* and rice CaMs were contained into the same group (Group I), while the *Solanaceous* CaMs were separated into two groups (Groups I and II), among which the group II was newly expanded (Figure 4). The *CaM* genes encoding Group II proteins carry three introns while those genes encoding Group I proteins contain only one (Figure 3), supporting the phylogenetic classification into two CaM groups. These data revealed that the *Solanaceous* species have evolved a new type of CaM during the evolution.

### Bioinformatics prediction of potential cis regulatory sequences for *SlCaM* gene expression

The 5′ upstream noncoding sequences of the *CaM* genes encoding identical CaM proteins are not well conserved (data not shown), suggesting that these genes may be differentially expressed in response to various stimuli, even though they encode identical proteins.

To obtain hints for how expression of the *CaM* genes may be regulated, potential cis-acting elements in upstream 1000 bp sequences of the *SlCaM* genes were analyzed. PLACE analysis revealed that *SlCaM* gene upstream sequences carry a variety of potential cis-acting elements, including binding sites for transcription factors that are regulated by hormones such as abscisic acid (ABA), gibberellin (GA), auxin, jasmonic acid (JA) and ethylene (ETH). The patterns of cis-acting elements differed significantly among the *SlCaM* genes. The *SlCaM1* gene promoter contained 15 elements that may respond to all these five hormones, while all other *SlCaMs* lacked elements responsive to one or several hormones. *SlCaM4* lacked cis-elements predicted to be responsive to auxin and ethylene, *SlCaM2*, *SlCaM5* and *SlCaM6* contained elements known to be responsive to two hormones, while *SlCaM3* carried only cis-elements responsive to auxin. In addition, all the *SlCaM* upstream sequences carry an abundance of W-box elements, suggesting that *SlCaM* expression may be regulated by the WRKY transcription factors (Table 2).

**Table 2:** **The *cis*-acting elements in *SlCaM *gene promoters**

**Regulator**	***Cis*-acting element**	**Code**	**Number of elements**					
			**SlCaM1**	**SlCaM2**	**SlCaM3**	**SlCaM4**	**SlCaM5**	**SlCaM6**
ABA	ABRELATERD	S000414	4			5		1
	ABRERATCAL	S000507	1					1
	DPBFCOREDCDC	S000292	1				1	
	MYCATRD22	S000174	2					
	PYRIMIDINEBOXH	S000298	1			1		
GA	TATCCAOSAMY	S000403	1			1		2
	PYRIMIDINEBO	S000298	1			1		
ETH	ERELEE4ERE	S000037	1	1				
Auxin	NTBBF1ARROL	S000273	2	2	2		1	
JA	T/GBOXATPIN2	S000458	1			2		
WRKY transcription factor	WBOXNTERF3	S000457	4	3	3	3	4	3
	WRKY71OS	S000447	10	3	3	11	9	6
	WBOXATNPR1	S000390	1		2	4	4	2

### Expression of *SlCaM* genes in plants was organ-specific

**Figure 5:**
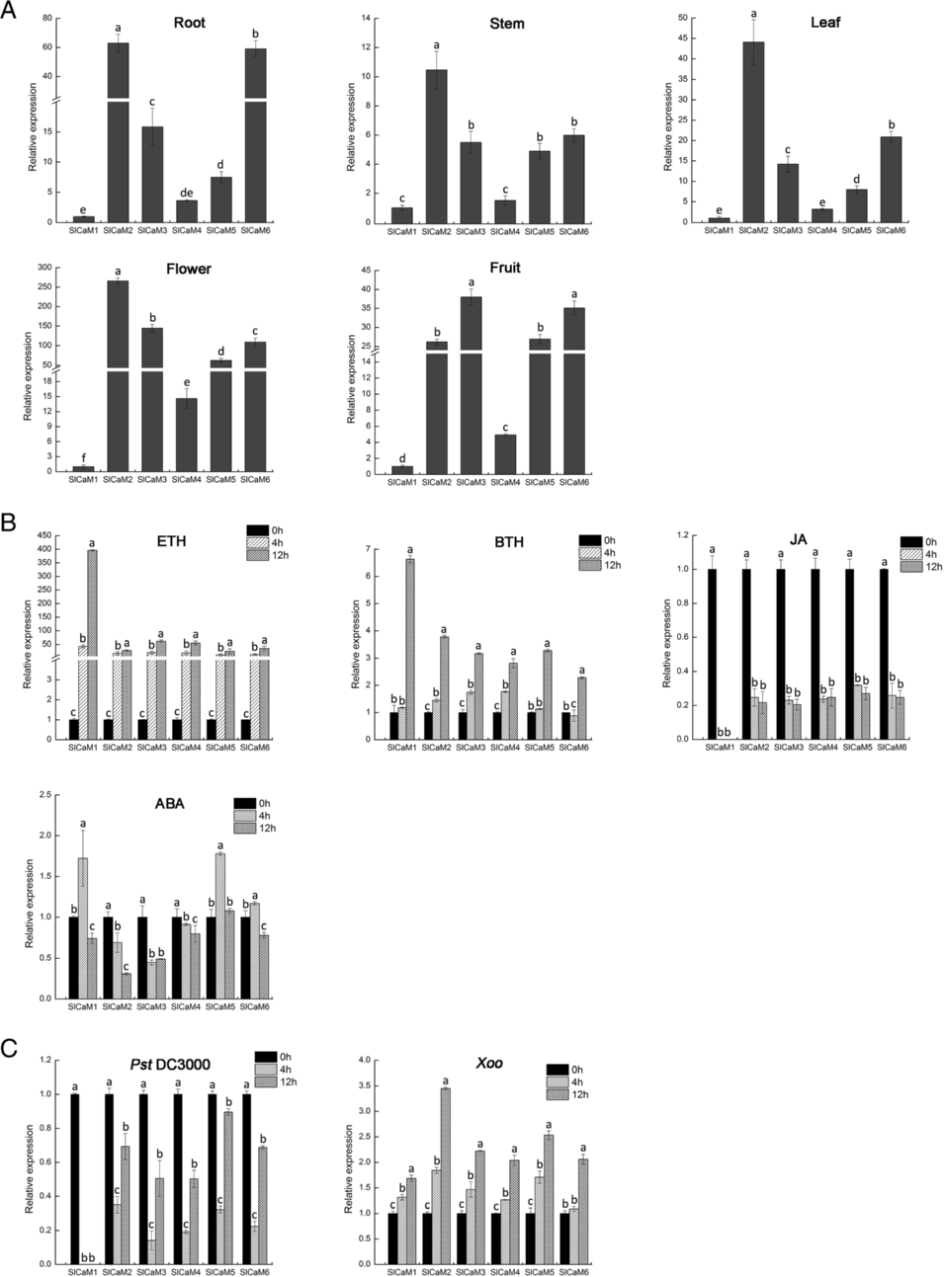
**Expression profiles of the *SlCaM *genes in plants.** Gene expression was analyzed by qRT-PCR. Expression levels relative to *SlCaM1* **(A)** and 0 h time-point **(B**, **C)** is shown. Significant difference between expression values within the target genes is indicated as different lowercase letters (P <0.05, DMRT). (**A**) Expression in different organs. **(B)** Expression in response to different hormone treatments. **(C)** Expression in response to pathogen inoculation with *Pst* DC3000 and *Xoo*.

The six *SlCaM* genes encode four CaM isoforms (Figures 1 and 2). To examine whether these genes are differentially expressed in developing plants and thus potentially harboring differential physiological functions, qRT-PCR analysis was conducted using *SlCaM* gene specific primers (Table 3). The expression patterns of *SlCaM* genes in roots, stems, leaves, flowers and fruit organs are shown in Figure 5A. Results of qRT-PCR expression analysis revealed that all *SlCaM* genes were constitutively expressed in all organs examined. However, the levels of expression were distinct in the various organs. Among the *SlCaMs*, *SlCaM1* displayed the lowest expression level in all organs. *SlCaM4* also showed relatively low expression in all organs except flowers. *SlCaM2* exhibited the highest transcript accumulation level in all organs except fruits. *SlCaM3* was expressed modestly in roots, stems and leaves, but was highly expressed in fruits and flowers. Expression profiles of *SlCaM5* were similar to that of *SlCaM3*, although the absolute expression level was relative lower. *SlCaM6* was expressed highly in roots, fruits and flowers, and modestly in stems and leaves. Thus, *SlCaM3*, *SlCaM4* and *SlCaM5* genes, which encode an identical protein, showed different expression levels in the various organs (Figure 5A). Generally speaking, the *SlCaM* genes were expressed highest in flowers and lowest in stems (Figure 5A).

**Table 3:** Primers used for qRT-PCR gene expression analyses

**Primer name**	**Sequence (5′ → 3′)**
SlCaM1-F	TTCCATTTCAAAGTATCTC
SlCaM1-R	GGTCCCATTTCCATCAGCA
SlCaM2-F	CTGATGAAGAAGTCGATGAGATG
SlCaM2-R	AGACAAGAGCCTACCCAATGA
SlCaM3-F	CACAACTTTCTTCTTCTCCC
SlCaM3-R	TCCCAACGACCTCATCACAG
SlCaM4-F	CATTTTCACACACACACTA
SlCaM4-R	TCAGCCTCAGTTGGGTTCT
SlCaM5-F	ATGACAAACCTAGGCGAGAAGC
SlCaM5-R	AACAAGAACGAATACACAAGAATC
SlCaM6-F	ATGAGATGATCCGAGAGG
SlCaM6-R	AGGCCACTAATATACTTGAACC
TRV2 2b-F	ATGCACGAATTACTTAGGAAG
TRV2 2b-R	GGTAACCTTACTCACAGAAT
TRV1 Rep-F	ATCTCAAGTTGATTTGAGGTT
TRV1 Rep-R	TGATCTCTTTGCTTACATCGT
SlPR1-F	TCTTGTGAGGCCCAAAATTC
SlPR1-R	ATAGTCTGGCCTCTCGCACA
SlPR5-F	AATTGCAATTTTAATGGTGC
SlPR5-R	TAGCAGACCGTTTAAGATGC
SlCNGC17-F	CATCATATCCACAGTCTGAT
SlCNGC17-R	CTCATTTGAACCAATGAAGT
SlCNGC18-F	CTGAAGATGAAGATGAAGAT
SlCNGC18-R	GTTTCTGTCATCACGACTA
SlGSTF2-F	CGGATGGGTGAGTATCGCGTTG
SlGSTF2-R	TGTGACACAGGAGTTAGGAAAC
SlUEP-F	ATGCAGATCTTCGTGAAAACCC
SlUEP-R	TCAATCGCCTCCAGCCTTGTTG

### Expression of *SlCaM* genes was highly responsive to diverse stimuli

To gain further information about potential differential gene functions, *SlCaM* expression patterns in response to various plant hormones were investigated. Expression data demonstrated that *SlCaM2* to *SlCaM6* showed similar expression patterns in response to ETH, BTH and JA, which was, however, different from that of *SlCaM1* in expression level (Figure 5B). Expression of *SlCaM2* to *SlCaM6* was dramatically up-regulated with ETH treatment as early as 4 hpt (hours post treatment) and then maintained the elevated expression level, moderately up-regulated with BTH treatment at 12 hpt, while strongly down-regulated with JA treatment after 4 hpt. Expression of *SlCaM1* showed a similar trend but had a significant different magnitude in response to these three hormones in comparison with *SlCaM2* to *SlCaM6*. *SlCaM1* was very highly up-regulated at 12 hpt by ETH and BTH treatments and down-regulated to undetectable levels at 4 hpt by JA treatment. Regarding ABA treatment, the six *SlCaM* genes exhibited different responses, although the alteration magnitude was generally not as large as that in response to other tested hormones. Expression of *SlCaM2* to *SlCaM4* was down-regulated, whereas *SlCaM1*, *SlCaM5* and *SlCaM6* expression was up-regulated at 4 hpt and then down-regulated at 12 hpt (Figure 5B). These data indicate that SlCaMs might play different roles in regulation of various hormone-regulated biological processes.

To determine whether there are roles for *SlCaM* gene function in disease resistance, gene expression in response to a set of pathogens was examined. Expression of *SlCaM* genes varied in response to inoculation with different pathogens (Figure 5C). Expression of *SlCaM2* to *SlCaM6* was significantly reduced at 4 h and then increased at 12 h after inoculation with *Pseudomonas syringae* pv. *tomato* DC3000 (*Pst* DC3000), whereas expression of *SlCaM1* was down-regulated to undetectable levels since 4 h after inoculation (Figure 5C). However, expression of all *SlCaM* genes was continuously up-regulated after inoculation with *Xanthomonas oryzae* pv. *oryzae* (*Xoo*). Among the *SlCaM* genes, expression of *SlCaM2* was most induced at 12 h after Xoo inoculation (Figure 5C). These data indicate that SlCaMs might be involved in regulation of plant disease resistance.

Collectively, in general, *SlCaM1* expression was much more sensitive to hormone treatment and pathogen inoculation in comparison with expression of the other *SlCaMs*.

### Silencing of *SlCaM2* and *SlCaM6* in tomato plants reduced resistance to TRV and *Pythium aphanidermatum*, but not to *Pst* DC3000 and *Xoo*

**Figure 6:**
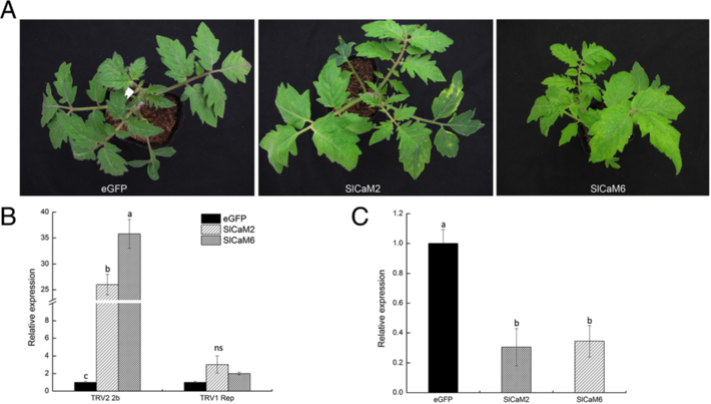
**Silencing of *SlCaM2 *and *SlCaM6 *in tomato plants reduced resistance to *Tobacco rattle virus *(TRV). (A)** Phenotypes of the *SlCaM* gene-silenced plants. Gene silencing analyses were performed using TRV-based vectors. TRV viral symptoms were more severe in *SlCaM* gene-silenced plants compared with the eGFP-control plants. Photographs were taken 3 weeks post agro-infiltration. **(B)** Detection of transcripts of the TRV_1_ replicase and TRV_2_ 2b genes in silencing plants by qRT-PCR. **(C)** Evaluation of gene silencing efficiency. Expression levels of the *SlCaM2* and *SlCaM6* genes in tomato plants were examined by qRT-PCR. Significant difference between expression values within the target genes is indicated as different lowercase letters (P <0.05, DMRT), while no significant difference as “ns”.

To understand the function of SlCaMs in plant disease resistance, virus-induced gene silencing (VIGS) was performed for *SlCaM2* and *SlCaM6*. A vector containing a fragment of eGFP was used as control in agro-infiltrated plants [26]. Three weeks post agro-infiltration, plants treated with eGFP-control showed no or only very weak mosaic symptoms in some leaves. However, most of the *SlCaM2*- and *SlCaM6*-silenced tomato plants displayed obvious mosaic and yellowing symptoms, which were most severe in the *SlCaM6*-VIGS plants (Figure 6A). Gene expression analysis revealed that transcripts of TRV_1_ replicase and TRV_2_ 2b genes accumulated over 2 and 25 times higher in these plants compared with eGFP-control plants (Figure 6B).

To check whether the symptoms and viral gene transcript accumulation levels correlated with silencing of *SlCaM2* and *SlCaM6* genes, transcripts of these genes in the agro-infiltrated plants were quantified with qRT-PCR. Result showed that *SlCaM2* and *SlCaM6* transcripts in the VIGS-treated plants dropped to about 30% of that in eGFP-control plants (Figure 6C). These results demonstrated that silencing of the *SlCaM2* and *SlCaM6* genes resulted in TRV viral symptoms and higher level of virus accumulation. Together, these data indicate that *SlCaM2* and *SlCaM6* may have a role in positively regulating tomato resistance to TRV.

**Figure 7:**
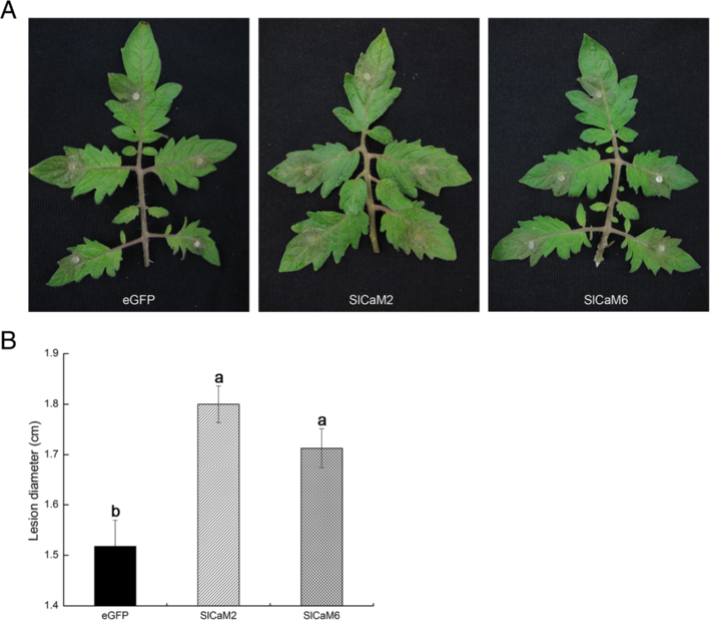
**Silencing of *SlCaM2 *and *SlCaM6 *in tomato plants reduced resistance to *Pythium aphanidermatum*. (A)** Disease symptoms of the SlCaM gene-silenced plants after inoculation with *P. aphanidermatum*. Photographs were taken 30 h post inoculation. **(B)** Statistical analysis of disease severity. Lesion diameter was recorded and statistically analyzed for all plants. Significant difference between lesion diameter of the plants is indicated as different lowercase letters (P <0.05, DMRT).

To further understand role of SlCaMs in disease resistance, the silenced tomato plant**s** were inoculated with the host pathogens *Pst* DC3000 and *Pythium aphanidermatum* and the nonhost pathogen *Xoo*, and resistance was evaluated. Nonhost resistance to *Xoo* in silenced plants was similar to that in eGFP-control plants, as both plants initiated hypersensitive response necrosis in infiltrated areas at 12 hpi, and showed complete tissue collapse within 24 hpi (Additional file 3A). Resistance to *Pst* DC3000 was also not altered significantly in silenced plants when compared with the eGFP-control plants. All genotypes showed necrosis at 12 hpi and died at 36 hpi (Additional file 3B). However, when inoculated with *P. aphanidermatum*, necrotic symptoms of the leaves of *SlCaM*-silenced plants were significantly more severe than that of eGFP-control plants (Figure 7A). The lesions in the silenced plants, 1.7 ~ 1.8 cm in diameter, were obviously larger in size than those in control plants, 1.5 cm in diameter (Figure 7B). This result revealed that the *SlCaMs* are necessary for enhanced resistance to *P. aphanidermatum* in tomato plants*.*

### Silencing of *SlCaM2* and *SlCaM6* in tomato plants altered expression of signaling and defense-related genes

**Figure 8:**
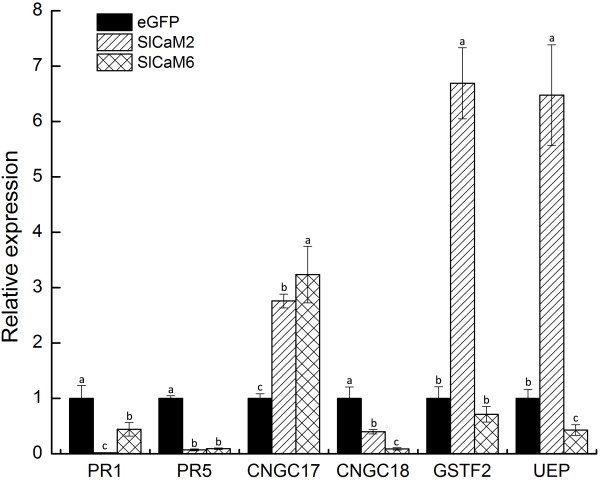
**Expression profiles of *SlPR*, *SlCNGC*, *SlUEP *and *SlGST *genes in *SlCaM*-silenced tomato plants.** Expression of the genes in tomato plants were examined by qRT-PCR with gene-specific primers listed at Table 3. Significant difference between expression values within the target genes is indicated as different lowercase letters (P <0.05, DMRT).

To probe the molecular mechanisms by which SlCaMs regulate plant resistance, we quantified the expression of two pathogenesis-related (PR) genes *PR1* and *PR5*, *SlCNGC17* and *SlCNGC18* which encode CNGC type calcium channel proteims, one glutathione transferase (GST) gene *SlGSTF2,* and an ubiquitin extension protein (UEP) gene by qRT-PCR. Silencing of both *SlCaM* genes significantly reduced the expression of *PR* genes and *SlCNGC18*, but increased that of *SlCNGC17*. In *SlCaM2*-silenced plants, the expression of *PR1*, *PR5* and *SlCNGC18* decreased by 98%, 93% and 60%, respectively, while that of *SlCNGC17* increased by 2.7 times. In *SlCaM6*-silenced plants, the expression of *PR1*, *PR5* and *SlCNGC18* lowered by 56%, 91% and 91%, respectively, while that of *SlCNGC17*enhanced by 3.2 times. However, silencing of the two *SlCaM* genes had differential effects on the expression of *SlGSTF2* and *SlUEP*; silencing of *SlCaM2* resulted in strongly enhanced expression of both genes by over 6.5 times whereas *SlCaM6* silencing reduced expression of *SlGSTF2* by 29% and that of *SlUEP* by 57% (Figure 8). These data indicate that the regulation of cytosolic Ca^2+^ concentration, ubiquitylation and redox status may be involved in *SlCaM*-mediated disease resistance, and *SlCaM2* and *SlCaM6* might employ different molecular mechanisms to regulate disease resistance.

## Discussion

To begin to understand the roles of CaM, the conserved eukaryotic Ca^2+^ sensor, in plants, we systemically identified the complete *CaM* gene families in the *Solanaceous* species tomato, potato and *N. benthamiana*. Previously, 5 full-length and 3 partial potato *CaM* genes were identified through screening a potato stolon tip cDNA library with a chicken *CaM* as probe [9,27]. Among the five full-length potato *CaMs*, *PCAM5*, *PCAM6*, *PCAM7*and *PCAM8* encode an identical CaM sequence, while *PCaM1* encodes another CaM isoform. Thus these five genes encode for two distinct potato CaM isoforms. In the present study, we performed genome-wide analysis for potato *CaM* genes and identified 4 full-length *StCaM* genes that we named *StCaM1* to *StCaM4*. These 4 genes encode 3 different StCaM isoforms because *StCaM2* and *StCaM3* encode identical proteins (Figures 1 and 2). StCaM1 is identical in sequence to PCaM5/6/7/8, while StCaM2/3 and StCaM4 are novel isoforms (Table 1, Figure 3).

Because of the large number of genes encoding proteins with sequence relatedness to CaM, there have been attempts to define canoical CaMs and distinguish them from calmodulin-like (CML) proteins. CaM is one of the most conserved proteins in eukaryotes. For example, the protein sequence identity between vertebrate and plant CaMs is near 90%. In this study, we found that all true CaMs of tomato, potato and *N. benthamiana* had amino acid sequence identity percentages over 91%, and the sequences of CMLs differed more significantly from the canonical CaMs with the identity percentages of lower than 80% (Table 1, Additional file 2, Figures 1 and 2). In addition, CaM is 149 amino acid in lengh and carries two pairs of EF-hand motifs, as exemplified by the *Solanaceous* CaMs identified in this study. We followed the criteria used in a previous analysis of the *CaM* and *CML* gene families of *Arabidopsis*[5] and identified CaMs as those proteins should include those that are composed of about 149 amino acids, harbor two pairs of EF-hand motifs, and share over 90% amino acid sequence identity with known canonical CaMs. Proteins that resemble CaM in that they have approximately 149 amino acids and four EF-hand motifs, but share less than 90% amino acid identity are defined as CaM-like (CML) proteins (Additional file 2). Using these criteria, some previously reported CaMs, such as NtCaM13 [8] and ScaM-4 and SCaM-5 [10], are not true CaMs, but are likely more appropriately referred to as CMLs.

A very unusual characteristic of CaM gene families is that a small set of genes, usually two to four, encode identical protein isoforms [4,5]. In the *Solanaceous* species used in this study, tomato *SlCaM3/4/5*, potato *StCaM2/3*, *N. benthamiana NbCaM1/2/3/4* and *NbCaM5/6* all encode an identical CaM isoform (Table 1, Figures 1 and 2). The phenomenon of multiple genes encoding identical CaM isoforms has been described in previous studies; *Arabidopsis AtCaM2/3/5*, *N. tabacum NtCaM3/4/5/6/7/8/11/12*, rice *OsCaM1-1/2/3*, soybean *GmCaM1/3* all share CaM isoforms [6-8,10]. Natural selection is likely acting to keep these protein sequences conserved because the encoding genes are not identical in sequence. The genes differ moderately in the coding sequences (mainly in the third nucleotides, wobble base, of the amino acid coding triplets, Figure 2), bear distinct 5′ upstream sequences (sequence data not shown), carry distinct introns with various sizes and sequences, and are located on different chromosomes (Table 1). This strong conservation argues that CaM plays critical roles in plant biological processes and that plants may need more than one copy of the *CaM* gene to execute essential functions. In addition, this phenomenon may also reflect a strategy of plants to evolve functional gene paralogs. These genes can be gained through sequence exchange among chromosomes. It is likely that all the *CaM* gene copies are functional because of the strong sequence conservation. However, it is likely that the different genes may respond differentially to stimuli since they contain different upstream sequences and introns. As an example, we found that tomato *SlCaM3/4/5* genes were expressed at different levels in plant tissues and in response to pathogens (Figure 5). The multiple *CaM* genes, which encode identical protein isoforms may be related to similar situations in which there are gene families that encode similar, but not identical proteins, such as those involved in disease resistance (R) [28,29] and encoding GST [30]. In cases where multiple genes encode closely related proteins, tandem gene arrangement and/or gene cluster(s) nearby on the same chromosome may facilitate the evolution of genetic diversity [28-30].

Another intriguing finding in this study is that *Solanaceous* species seem to have evolved one novel group of *CaM* genes when compared with *Arabidopsis* and rice. Phylogenetic analysis reveals that all *Arabidopsis* and rice CaM protein isoforms form one clade; however, the CaMs of *Solanaceous* species separated into two groups, one of them belong to the same clade as that of *Arabidopsis* and rice CaMs, while the other group members are unique (Figure 4). This classification of CaMs is supported not only by the amino acid sequence similarity but also by the gene structure data. All group I *CaM* genes contain a single intron, while group II *CaMs* have three introns (Figure 3). These data suggest the possibility that the different groups of CaMs may play distinct roles in plant biological processes.

Roles for plant CaMs in growth, development and stress resistance have been widely discussed. However, a function for CaMs in plant disease resistance remains not fully explored. In this study, we found that silencing of two *SlCaM* genes significantly altered signaling and defense-related gene expression, and reduced the resistance in tomato to TRV and the important oomycete pathogen *Pythium aphanidermatum* (Figures 6, 7 and 8). Therefore, the SlCaMs are important in viral and oomycete resistance. The molecular mechanisms by which CaMs may regulate disease resistance are unknown. We found in this study that silencing of *SlCaM2* and *SlCaM6* genes significantly reduced the expression of *SlCNGC18*, but increased that of *SlCNGC17*, and differentially altered the expression of *SlGSTF2* and *SlUEP* genes (Figure 8). These results provide a hint that regulation of cytosolic Ca^2+^ concentration, ubiquitylation and redox status may be involved in SlCaM-mediated disease resistance, and *SlCaM2* and *SlCaM6* might employ different molecular mechanisms to regulate disease resistance.

## Conclusions

The *CaM* gene families in *Solanaceous* species tomato, *N. benthamiana* and potato were identified. Tomato, potato and *N. benthamiana* genomes contain multiple *CaM* genes of high sequence conservation. *Solanaceous* species has evolved one new group of CaM genes, with distinct gene structure. Whether the different CaM groups play distinct roles remains to be analyzed.

Reduced expression of *SlCaM* genes *SlCaM2* and *SlCaM6* impaired resistance of tomato to TRV and the important oomycete pathogen *Pythium aphanidermatum*, demonstrating that these SlCaMs play important roles in plant disease resistance to a variety of pathogens. Finally, our results suggest that *SlCaM2* and *SlCaM6* may employ different molecular mechanisms to regulate disease resistance.

## Methods

### Identification of *CaM* genes in *Solanaceous* species

To find *CaM* genes in *Solanaceous* species, all four *Arabidopsis* and three rice CaM protein sequences were collected trough searching the genome sequence databases TAIR (The Arabidopsis Information Resource, http://www.arabidopsis.org/) and Rice Genome Annotation Database (http://rice.plantbiology.msu.edu/). All retrieved AtCaM and OsCaM protein sequences were used to TBLASTN search the genome databases of *Solanaceous* species including tomato, potato and *Nicotiana benthamiana* in Solanaceae Genomics Network (http://solgenomics.net/). All retrieved non-redundant sequences were collected, and subjected to domain analysis by using the Pfam (http://pfam.sanger.ac.uk/) and Conserved Domain Database (CDD) (http://www.ncbi.nlm.nih.gov/cdd) programs. These sequences were compared with *Arabidopsis* canonical CaM protein AtCaM2 using ClustalX 2.01 program [31] with default settings and were viewed by GeneDoc. Those containing two pairs of EF-hand motifs, displaying amino acid sequence identity of over 90% to AtCaM2 and having a size of about 149 amino acids were recognized as CaM proteins. CaMs in a given species were named in accordance with sequence similarity to *Arabidopsis* CaMs.

### Phylogenetic and gene structure analyses of *CaM* genes

*CaMs* from different plant species were aligned by using ClustalX 2.01 program [31] with default settings. The un-rooted phylogenetic trees were constructed based on alignments using MEGA 5.0 [32] with the maximum likelihood (ML) method. The bootstrap analysis was carried out setting up 1000 replicates. Exon-intron structure analyses were carried out using the Gene Structure Display Server (GSDS) program with default settings [33].

### Bioinformatics prediction of regulators of *SlCaM* gene expression

The upstream 1000 bp sequence of *SlCaM* genes were searched for a variety of cis-acting elements by ‘Signal Scan Search’ program in the PLACE database (http://www.dna.affrc.go.jp/PLACE/).

### Construction of the virus induced gene silencing (VIGS) constructs

The coding regions of the *SlCaM* genes are highly conserved. To specifically silence a target gene member, a gene-specific 3′ UTR sequence of each *SlCaM* gene was inserted into TRV-based VIGS vector pYL156 according to the following procedure. A 171 bp 3′ UTR fragment of *SlCaM2* gene (Solyc10g081170.1.1) was amplified by PCR from tomato using primers VSlCaM2-F (gcgaattcTTCCATTATCCTCTTGTTACA, a *Eco*RI site was introduced) and VSlCaM2-R (ttggatccGTAGAGATCACACCACTCATAC, a *Bam* HI site was introduced), while a 286 bp fragment of *SlCaM6* gene (Solyc03g098050.2.1) was amplified by primers VSlCaM6-F (gcgaattcTGACTTTAAGATTCTGTTAGCT, a *Eco*RI site was introduced) and VSlCaM6-R (ttggatccGATATTACCAATGAACTATCTA, a *Bam* HI site was introduced). The resulting PCR product was cloned into pYL156 with *Eco* RI/*Bam* HI, and confirmed by sequencing. The recombinant constructs were transformed into *Agrobacterium tumefaciens* (strain GV3101) for VIGS analysis.

### VIGS manipulation procedure

VIGS analysis in tomato was conducted as described [34,35] except using pTRV_2_-eGFP instead for empty pTRV2 as a negative control vector so that the viral symptom can be repressed efficiently [26]. Briefly, agro-inoculi harboring pTRV_1_ and pTRV_2_-eGFP or pTRV_2_-SlCaM were vacuum infiltrated into cotyledons of seedlings just developing the first true leaves. The agro-inoculated plants were grown in a plant growth chamber at 21°C with a 16 h/8 h light/dark regime. Three weeks later, the plants were subjected to disease resistance analyses, and leaves were sampled to check the gene silencing efficiency and the accumulation of *TRV* gene transcripts by qRT-PCR with specific primers (Table 3).

### Plant materials for expression analysis

Tomato plants (cultivar Suhong2003) were grown in growth chambers at 28°C with a 16 h/8 h light/dark daily cycle. Different organs including roots, stems, leaves flowers and fruits were collected from 4-month-old tomato plants. Harvested organs were immediately frozen in liquid nitrogen and stored at -80°C.

For hormone treatment, leaves of 7- to 8-week-old tomato seedlings were sprayed with 100 μM abscisic acid (ABA), 10 mM ethephon (ETH), 200 μM jasmonic acid (JA), 350 μM benzothiadiazole (BTH), or sterilized water as control, respectively. Leaves were sampled at 4 h and 12 h post treatment for analysis of *SlCaM* gene expression.

For pathogen inoculation, the bacterial pathogens *Pseudomonas syringae* pv. *tomato* DC3000 (*Pst* DC3000) and *Xanthomonas oryzae* pv*. oryzae* (*Xoo*) were incubated overnight at 28°C on King’s medium B plates containing rifampicin (50 μg/mL) and kanamycin (50 μg/mL) and NA liquid medium respectively. The bacterial cells were collected by centrifugation and then diluted into suspensions to a concentration of OD_600_ = 0.002 and 0.5 using 10 mM MgCl_2_ buffer or sterilized ddH_2_O, respectively. The prepared bacterial solution (10 mM MgCl_2_ buffer or sterilized ddH_2_O as controls) was infiltrated into leaves of 7- to 8-week-old tomato plants. Samples were taken at 0 h, 4 h and 8 h after infiltration with *Xoo* and 0 h, 4 h and 12 h with *Pst* DC3000, respectively, for gene expression analysis.

### Gene expression analysis with real time PCR

Total RNA was extracted by Trizol regent (TAKARA, Japan) according to the manufacturer’s instructions. RNA was treated with DNase I (TAKARA, Japan) and reverse-transcribed into cDNA using the PrimeScript RT regent kit (TAKARA, Japan). The obtained cDNAs were used for gene expression detection analysis with real time quantitative PCR. RT-PCR was conducted in StepOne Real-Time PCR System (Applied Biosystems, USA) using SYBER Premix Ex Taq reagents (TAKARA, Japan) following the program: 95°C for 30 s, 95°C for 5 s and 60°C for 45 s for 40 cycles. To normalize the sample variance, *18S rDNA* gene was served as the internal control. Relative gene expression values were calculated using the 2^-△△Ct^ method. To ensure gene-specificity of RT-PCR, primers were designed according to the 5′ and 3′ UTR regions of the *SlCaM* genes. The primers used for gene expression analysis are listed at Table 3.

The experiments were conducted three times, each containing three replicates for all genes. For the statistical analysis of the gene expression data, ANOVA (analysis of variance) analysis was performed with SPSS software (Version 19.0, IBM, USA). Significance of the differences between mean values was determined with Duncan’s multiple range test (DMRT).

### Plant disease resistance analysis

The VIGS-treated plants were subjected to disease resistance evaluation. Bacterial pathogens *Pst* DC3000 and *Xoo* were performed as described above. The oomycete pathogen *Pythium aphanidermatum* were grown at 25°C on potato dextrose agar (PDA) medium for 2 d. PDA plugs of 3 mm in diameter were taken from the outside circle containing most actively young mycelia, and were put on the newly developed leaves of the VIGS-treated plants. After inoculation, the plants were maintained at high relative humidity for 2 d. Disease or HR symptoms were investigated. Size of the necrosis was recorded and photographs were taken accordingly.

Resistance to each pathogen was analyzed in at least six plants per experiment, and the experiment was repeated twice.

## Abbreviations

ABA: Abscisic acid; BTH: Benzothiadiazole; CaM: Calmodulin; CNGC: Cyclic nucleotide gated channel; ETH: Ethephon; GST: Glutathione transferase; JA: Jasmonic acid; NtCaM: *Nicotiana benthamiana* calmodulin; PR: Pathogenesis-related; Pst DC3000: *Pseudomonas syringae* pv. *tomato* DC3000; SlCaM: *Solanum lycopersicum* calmodulin; StCaM: *Solanum tuberosum* calmodulin; Xoo: *Xanthomonas oryzae* pv*. oryzae*; UEP: Ubiquitin extension protein; VIGS: Virus-induced gene silencing.

## Competing interests

The authors declare that they have no competing interests.

## Authors’ contributions

The project was coordinated by XZC. YZ and WL conducted the bioinformatics and phylogenetic analyses. YZ, YPX and JYC carried out the gene expression assays and VIGS analyses. YZ designed and performed the statistical analysis. XZC conceived of the study, and participated in its design and coordination. JB provided advice on protein classification. XZC, JB and YZ prepared the manuscript. All authors read and approved the final manuscript.

## Supplementary Material

Additional file 1 **Alignment profile of NbCaM (A), StCaM (B) and SlCaM (C) coding sequences.** The accession numbers for the *CaM* genes were listed at Table 1. Click here for file

Additional file 2 **CML, CDPK and other sequences retrieved from BLAST searches against SGN databases with *Arabidopsis *and rice CaM protein sequences.** Click here for file

Additional file 3 **Hypersensitive response symptoms in *SlCaM2*- and *SlCaM6*-silenced plants inoculated with bacterial pathogens *Pst* DC3000 and *Xoo. ***Plants infiltrated with *Agrobacterium* suspensions carrying an eGFP control vector were served as control plants. Photographs were taken at 36 h post *Pst* DC3000 inoculation (A) and 48 h post *Xoo* inoculation (B). Click here for file
