# Supplementary figures and images for: Genome-wide identification and functional analyses of calmodulin genes in Solanaceous species
Source: BMC Plant Biol. 2013 Apr 27;13:70. doi: 10.1186/1471-2229-13-70 (PMC3751459; doi:10.1186/1471-2229-13-70)

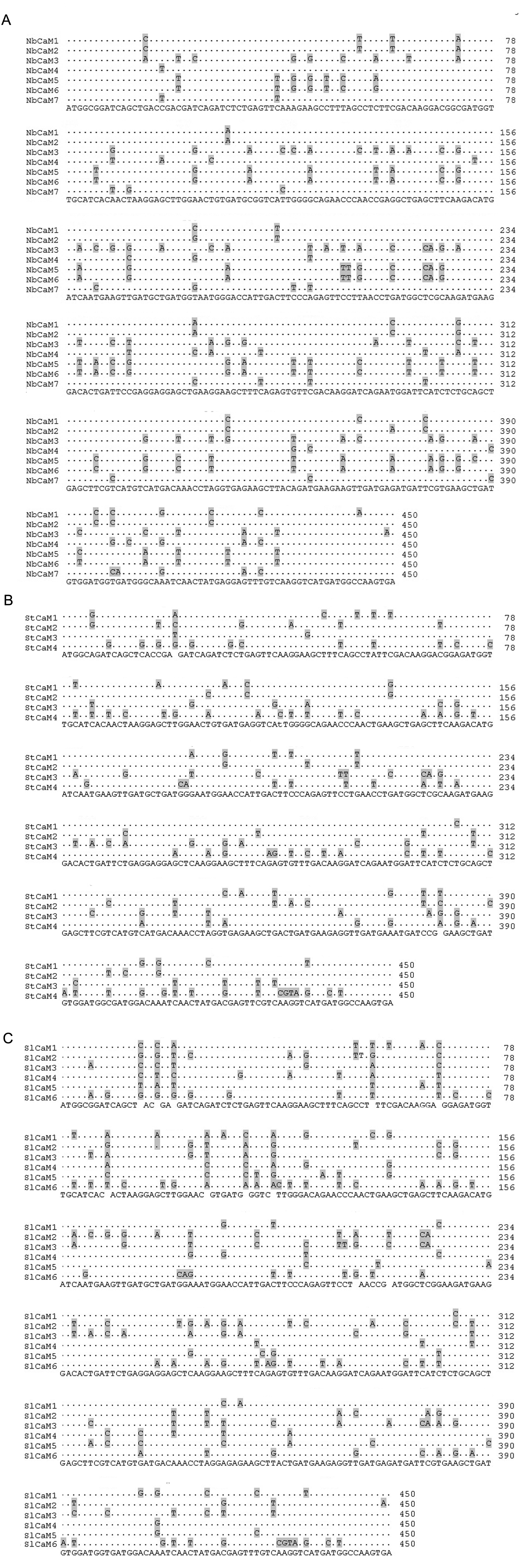

Supplement: Additional file 1 — Alignment profile of NbCaM (A), StCaM (B) and SlCaM (C) coding sequences. The accession numbers for the CaM genes were listed at Table 1. [file 1471-2229-13-70-S1.jpeg]

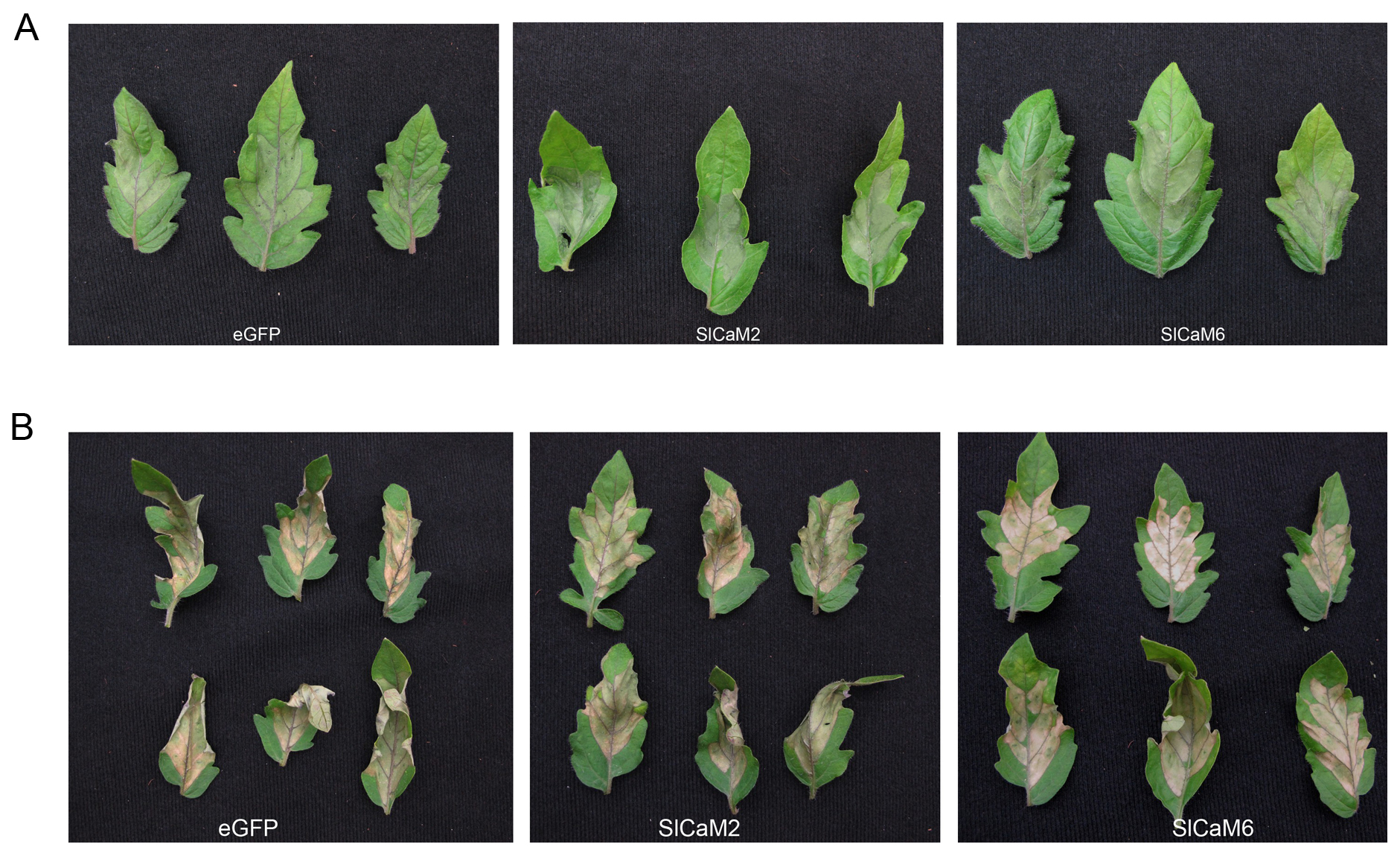

Supplement: Additional file 3 — Hypersensitive response symptoms in SlCaM2- and SlCaM6-silenced plants inoculated with bacterial pathogens Pst DC3000 and Xoo. Plants infiltrated with Agrobacterium suspensions carrying an eGFP control vector were served as control plants. Photographs were taken at 36 h post Pst DC3000 inoculation (A) and 48 h post Xoo inoculation (B). [file 1471-2229-13-70-S3.jpeg]
